# Complete genome sequences of three *Pseudomonas aeruginosa* phages of the genus *Phikmvvirus*

**DOI:** 10.1128/mra.01010-24

**Published:** 2025-02-06

**Authors:** Tracey L. Peters, Olga A. Kirillina, Martin O. Georges, Katie R. Margulieux, Kevin A. Burke, Nino Mzhavia, Paphavee Lertsethtakarn, Lillian A. Musila, Andrey A. Filippov, Mikeljon P. Nikolich

**Affiliations:** 1 Institute for Modeling Collaboration and Innovation, University of Idaho, Moscow, Idaho, USA; 2 Wound Infections Department, Bacterial Diseases Branch, Walter Reed Army Institute of Research, Silver Spring, Maryland, USA; 3 Department of Emerging Infectious Diseases, Walter Reed Army Institute of Research-Africa, Nairobi, Kenya; 4 Department of Bacterial and Parasitic Diseases, Walter Reed Army Institute of Research-Armed Forces Research Institute of Medical Sciences (WRAIR-AFRIMS), Bangkok, Thailand; Loyola University Chicago, Chicago, Illinois, USA

**Keywords:** *Pseudomonas aeruginosa*, bacteriophages, *Phikmvvirus*, complete genomes, therapeutic candidates

## Abstract

We describe the genomes of three lytic *Pseudomonas aeruginosa* phages of the genus *Phikmvvirus*. The genomes of phages vB_Pae4841-AFR43, vB_Pae10145-KEN1, and vB_Pae9718-KEN10 consist of 43,426, 43,406, and 43,118 bp, with 62.4%, 62.3%, and 62.2% GC content, contain 63, 66, and 64 coding sequences, respectively, and no tRNA genes.

## ANNOUNCEMENT

Globally proliferating multidrug-resistant *Pseudomonas aeruginosa* infections compel the use of phages as adjunct antibacterials, with demonstrated efficacy in humans (1–3). Walter Reed Army Institute of Research is harvesting *P. aeruginosa* phages on four continents for incorporation into therapeutic cocktails. Here, we describe the genomes of phages vB_Pae4841-AFR43 (AFR43), vB_Pae10145-KEN1 (KEN1), and vB_Pae9718-KEN10 (KEN10).

Phages were isolated from fresh water collected on 6 October 2020 in Bangkok, Thailand (AFR43, GPS coordinates 13.767744, 100.536810) and urban sewage samples on 19 February 2021 in Nairobi, Kenya (KEN1 and KEN10, GPS coordinates −1.3180522, 36.8031984). Enrichment was performed as previously described (4) using *P. aeruginosa* strains MRSN 4841 (AFR43), ATCC 10145 (KEN1), and MRSN 9718 (KEN10), producing phage plaques on double-layer agar plates. Three rounds of single-plaque isolation ensured purity. Phages were propagated in Heart Infusion Broth, and genomic DNA was extracted using the QIAamp DNA Mini Kit (Qiagen, Germantown, MD) (4). Libraries were prepared using the KAPA HyperPlus Kit (Roche Diagnostics, Indianapolis, IN) and sequenced on an Illumina MiSeq (Illumina, Inc., San Diego, CA) using MiSeq Reagent Kit v3 (600 cycles, 300 bp reads). The quality of 192,484 (AFR43), 458,490 (KEN1), and 645,934 (KEN10) paired end reads was evaluated and trimmed using Fastp (5) v0.22.0. Genomes were assembled with Unicycler (6) v0.5.0, and termini were identified using PhageTerm (7) v3.0.1. Phage lifestyle was predicted with BACPHLIP (8) v0.9.6, and protein coding sequences (CDSs) annotated using the Pharokka pipeline (9–19) v1.6.0. Amino acid sequence similarity searches were performed using DIAMOND (20, 21) v.2.1.8. Default parameters were used for all analyses.

**Fig 1:**
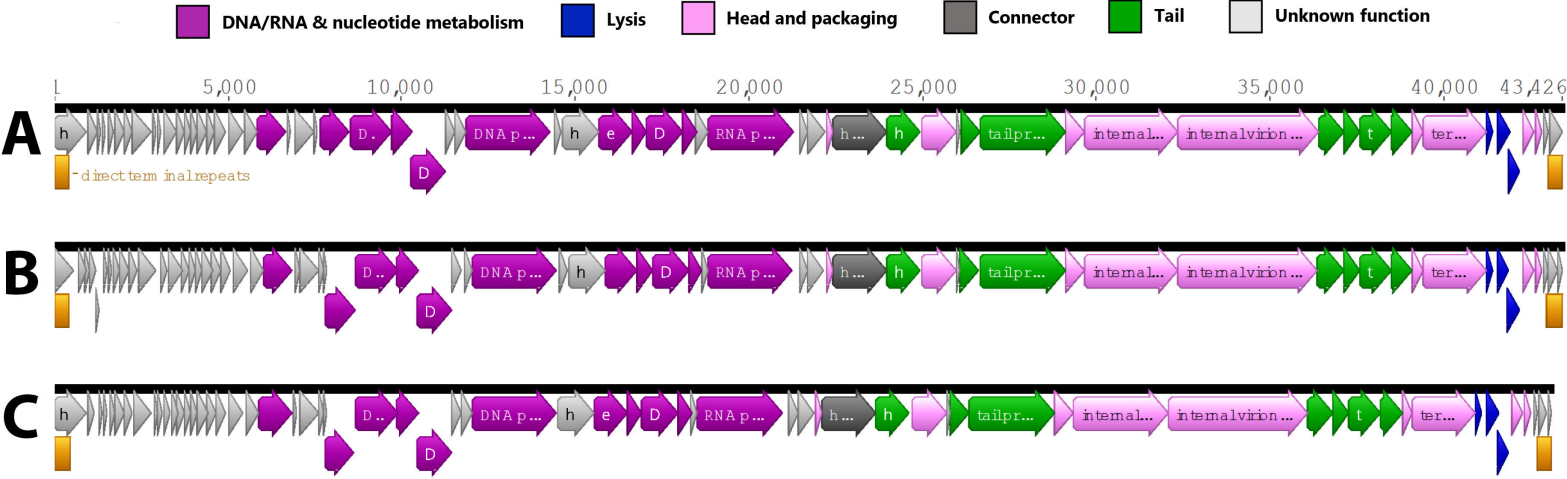
Genome organization of three *Phikmvvirus* phages isolated in Thailand and Kenya. (A) AFR43; (B) KEN1; and (C) KEN10. The genome maps were prepared in Geneious v2024.0.5 and edited in Inkscape v1.3.2.

Average read coverages for phages AFR43, KEN1, and KEN10 were 816, 2,316, and 2,129, the genomes were 43,426, 43,406, and 43,118 bp long, with 62.4%, 62.3%, and 62.2% GC content, contained 63, 66, and 64 predicted CDSs, and direct terminal repeats of 418, 420, and 440 bp, respectively (see Fig. 1). Using Mash v2.2.2 alignment (19) against the INPHARED database (18), these phages were classified into the family *Autographiviridae*, subfamily *Krylovirinae*, genus *Phikmvvirus*. Blastn 2.16.0+ (10.1089/10665270050081478) alignments showed 80.8%–95.8% whole-genome average nucleotide identity (wgANI) (https://doi.org/10.1038/nbt.4306) compared to 16 *Phikmvvirus* reference genomes (https://ictv.global/taxonomy, retrieved 22 August 2024) with the exception of a 76.7% wgANI between phage Ken10 and phage LDK16 (NC_009935), which is below the cutoff of ≥80.75%. Phages of this group have podovirus morphology, are lytic, usually produce large clear plaques, often have broad activity, and were included in some commercial therapeutic phage cocktails used in Russia and Georgia (22–26). The closest relative of AFR43, KEN1, and KEN10 (92%–94% identity) was *Pseudomonas* phage RLP that has shown efficacy against *P. aeruginosa* bacteremia in mice (25). No tRNA genes were identified in these three phages, which is typical of the group (22, 25, 26).

BACPHLIP scored AFR43, KEN1, and KEN10 genomes at 100% for a virulent life cycle (8). No homology was found between putative proteins of these phages and products associated with lysogenicity, gene transfer, and bacterial proteins including antibiotic resistance determinants (14) and virulence factors (15). Thus, AFR43, KEN1, and KEN10 are most likely lytic phages and promising therapeutics.

## Data Availability

The AFR43, KEN1, and KEN10 genome BioProject accession number is PRJNA1083498, BioSample accession numbers are SAMN40272538, SAMN40272539, and SAMN40272540, GenBank accession numbers are PP501790, PP501791, and PP501792, and the NCBI Sequence Read Archive accession numbers are SRR28260741, SRR28260740, and SRR28260739, respectively.
